# Development of an Innovative Colorimetric DNA Biosensor Based on Sugar Measurement

**DOI:** 10.3390/bios13090853

**Published:** 2023-08-28

**Authors:** Maliana El Aamri, Yasmine Khalki, Hasna Mohammadi, Aziz Amine

**Affiliations:** Laboratory of Process Engineering & Environment, Faculty of Sciences and Techniques, Hassan II, University of Casablanca, B.P.146, Mohammedia 28806, Morocco; maliana.elaamri@etu.fstm.ac.ma (M.E.A.); yasmine.khalki@etu.fstm.ac.ma (Y.K.); hasna2001fr@yahoo.fr (H.M.)

**Keywords:** sugar reaction, colorimetric biosensor, DNA target, RNA target, intercalator detection

## Abstract

The development of biosensors for target detection plays a crucial role in advancing various fields of bioscience. This work presents the development of a genosensor that exploits the colorimetric phenol—sulfuric acid sugar reaction for the detection of DNA, and RNA as specific targets, and DNA intercalator molecules. The biosensor combines simplicity and reliability to create a novel bioassay for accurate and rapid analysis. A 96-well microplate based on a polystyrene polymer was used as the platform for an unmodified capture DNA immobilization via a silanization process and with (3-Aminopropyl) triethoxysilane (APTES). After that, a hybridization step was carried out to catch the target molecule, followed by adding phenol and sulfuric acid to quantify the amount of DNA or RNA sugar backbone. This reaction generated a yellow-orange color on the wells measured at 490 nm, which was proportional to the target concentration. Under the optimum conditions, a calibration curve was obtained for each target. The developed biosensor demonstrated high sensitivity, good selectivity, and linear response over a wide concentration range for DNA and RNA targets. Additionally, the biosensor was successfully employed for the detection of DNA intercalator agents that inhibited the hybridization of DNA complementary to the immobilized capture DNA. The developed biosensor offers a potential tool for sensitive and selective detection in various applications, including virus diagnosis, genetic analysis, pathogenic bacteria monitoring, and drug discovery.

## 1. Introduction

Analytical methods based on the quantitative determination of sugars plays a fundamental role in various applications of biosciences. The phenol–sulfuric acid method is widely employed for determination of sugars in oligosaccharides, glycoproteins, glycolipids, and even DNA [1,2,3]. This colorimetric method is recognized for its simplicity and reliability, making it a preferred choice for sugar analysis across multiple fields of study. The phenol–sulfuric acid method for sugar detection is a widely adopted approach due to its notable sensitivity and straightforwardness. Although alternative methods, such as those employing anthrone [4], orcinol [5], or resorcinol [6], may exhibit similar levels of sensitivity, they present distinct advantages and challenges. Anthrone-based assays are recognized for their sensitivity but often involve complex reaction conditions and the need for multiple reagents, potentially complicating experimental protocols. Orcinol-based methods offer good sensitivity but can be sensitive to reaction conditions, leading to variability in results. Resorcinol-based assays are relatively simple but may suffer from interferences due to the presence of other compounds. Furthermore, all these colorimetric methods require heating at least 90 °C for a few minutes (around 10 min). In contrast, the phenol sulfuric acid assay is an instantaneous reaction at room temperature. It offers a balance of sensitivity and simplicity, requiring fewer reagents and straightforward reaction conditions. This combination of attributes makes the phenol–sulfuric acid approach particularly convenient and user-friendly, thereby enhancing its applicability in various settings.

In the field of nucleic acid detection for in vitro assays, an array of biosensors based on chemiluminescent [7], electrochemical [8], electrochemiluminescent [9], fluorescent [10], and luminescent [11] biosensors has emerged, each harnessing distinct principles for sensitivity and specificity. However, colorimetric biosensors offer rapid and cost-effective detection methods that can be employed at the point of care, enabling real-time diagnosis of biomarkers and emerging viruses [12,13]. Indeed, various colorimetric biosensors have been developed to specifically detect and quantify nucleic acids, offering valuable insights into virus diagnostics, genetic analysis, and biotechnological applications [14,15,16,17]. These assays use diverse dyes or nanoparticles, serving as indicators [18,19,20], which undergo distinct color changes upon interaction with target DNA or RNA molecules. The detection principle often involves the formation of complexes or aggregates, leading to alterations in the absorbance spectrum. Such colorimetric detection strategies provide a promising avenue for sensitive and label-free nucleic acid analysis, presenting an attractive alternative to more complex and expensive analytical techniques.

Furthermore, biosensors play a crucial role in the sensitive detection of intercalator agents and genotoxic molecules by leveraging their ability to interact with DNA through intercalation, which hinders the DNA hybridization step or provides valuable insights into potential DNA damage and mutagenicity [21,22]. These specialized sensors are designed to recognize and bind to intercalating compounds that can insert themselves between the base pairs of DNA, leading to structural modifications [23]. As a result, the hybridization process, which involves the pairing of complementary DNA strands, is inhibited. By monitoring this interference, biosensors can provide valuable information about the presence and concentration of genotoxic substances in various samples [24]. Untiveros et al. developed an electrochemical biosensor using a Stem–Loop DNA probe for detecting DNA damage induced by the hybrid drug (7ESTAC01). This compound combines acridine and thiophene pharmacophores for anti-cancer activity. The resulting interaction and DNA damage caused by 7ESTAC01 were electrochemically quantified through differential pulse voltammetry (DPV), measuring oxidation signals of electroactive nucleic acids [25]. On the other hand, Lei et al. introduced an innovative electrochemical DNA biosensor utilizing pencil graphite electrodes, enhanced with polypyrrole/Ce-doped hexagonal nickel oxide nanodisk composites, to detect Abemaciclib (AMC) and ds-DNA molecules. Their approach involved DPV for the electrochemical detection of AMC [26]. The utilization of biosensors for this purpose holds great promise in environmental monitoring, medical diagnostics, and toxicological studies, enabling efficient and reliable detection of potentially harmful agents and contributing to the advancement of public health and safety.

In this study, our objective is to develop a colorimetric biosensor that exploits the sugar reaction for the detection of DNA target, RNA target, and intercalator molecules. Building upon the widely utilized phenol–sulfuric acid method, we aim to harness its simplicity and sensitivity to extend its applicability to target detection in nucleic acids and intercalator molecules. By integrating the sugar reaction with colorimetric indicators (phenol–sulfuric acid reagents), we seek to create a novel biosensing platform capable of accurate and rapid analysis of these important biomolecules. To achieve this, we followed a method similar to that described by Maliana et al. [12], where a sequence probe complementary to the target sequence was immobilized on the surface of 96-microplate wells. The sample containing the sequence target was then added to the prepared microplate and subjected to two washes. Subsequently, a phenol–sulfuric acid reagent was added to the microplate, resulting in the immediate appearance of an orange color. The absorbance of the colored complex was measured using a microplate reader at 490 nm. The obtained absorbance was directly correlated with the concentration of the target DNA or RNA. Hence, a higher concentration of the target DNA or RNA yielded a greater level of the colored complex. This principle enables the quantification of the target DNA or RNA using the developed biosensor, offering a potential tool for sensitive and specific detection in various applications. Furthermore, we also developed a colorimetric biosensor for the detection of intercalator agents that inhibit the hybridization of DNA complementary to the immobilized capture DNA on the 96-microplate wells.

## 2. Materials and Methods

### 2.1. Chemicals and Reagents

Adenosine 5′-triphosphate (ATP) disodium salt hydrate, Potassium hydroxide (KOH), phenol, DNA fish sperm, Curcumin, ribose, glucose, fructose, potassium chloride (KCl), glutaraldehyde (Glu), and ethanolamine (ETA) were bought from Merck, Darmstadt, Germany. (3-Aminopropyl) triethoxysilane (APTES) was obtained from ALFA AESAR, Lancashire, UK. Hydrochloric acid (HCl) was bought from VWR life science AMRESCO, Limerick, Ireland, while ethanol was acquired from Laurylab, Brindas, France. Sulfuric acid (H_2_SO_4_) and sodium phosphate dibasic (Na_2_HPO_4_, 12H_2_O) were bought from Solvachim (Casablanca, Morocco).

The chemicals necessary for preparing the phosphate-buffered saline (0.01 M of PBS with 2.7 mM of KCl and 137 mM of NaCl, pH 7.4) were purchased from Merck, Germany, and all reagents used were of analytical grade.

PBS served as the washing buffer.

HPLC pure oligonucleotides in lyophilized powder form were supplied by Eurofins Genomics, The Ulis, France. Oligonucleotide sequences are detailed in Table 1. Stock solutions of synthetic oligonucleotides were created in ultra-pure water, divided into aliquots, and stored at −20 °C.

### 2.2. Apparatus

Absorbance readings were taken using an ELx800 absorbance microplate reader (BioTek, Winooski, VT, USA) to assess the optical density of the generated colorimetric product at 490 nm. Analysis of the data was conducted using Gen5 software (V 1.0.4). Data analysis, artwork creation, and graphing were performed utilizing OriginPro8.

To conduct the characterization, a JEN-WAY UV-Vis double beam spectrophotometer (model 6850) from the UK, equipped with a matched 1.0 cm cell, was utilized.

### 2.3. Microplate-Based Phenol–Sulfuric Acid Method for Sugar Source Analysis

**Scheme 1:**
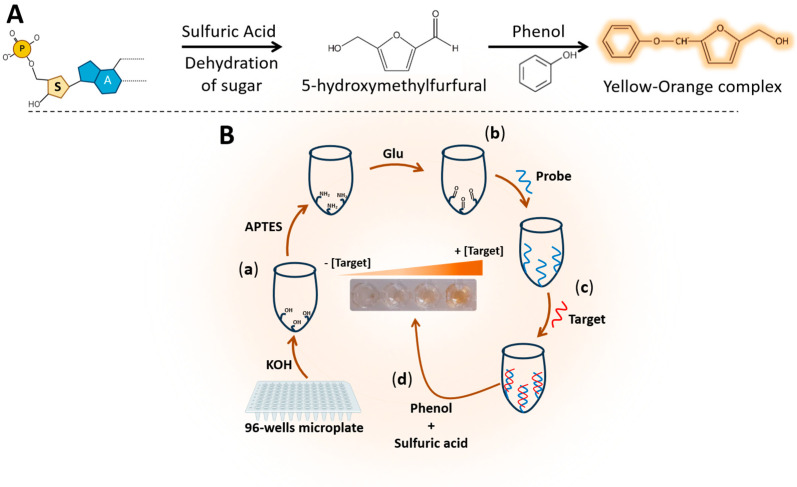
(**A**) Schematic overview of the phenol–sulfuric acid mechanism for the sugar DNA/RNA analysis. (**B**) Illustration of the colorimetric biosensor based on the phenol–sulfuric acid reaction for DNA or RNA detection.

The phenol–sulfuric acid reaction is well-established for its proficiency in detecting and quantifying sugars as shown by the mechanism in Scheme 1A. This reaction involves the combination of phenol and sulfuric acid, resulting in the creation of a stable-colored complex. Notably, it has been reported that phenol undergoes sulfonation in situ during this reaction, leading to the production of a sulfonic acid derivative [27]. This sulfonic acid derivative plays a crucial role in generating the characteristic color utilized for sugar analysis, which is typically measured at 490 nm. To initiate the reaction, 100 µL of the sugar solution was added to a microplate, followed by the addition of 100 µL of concentrated sulfuric acid, and 10 µL of phenol (120 mg/mL). A distinctive yellow-orange color was developed, indicative of the presence of sugar. The reaction was carried out at room temperature.

### 2.4. Construction of the Phenol–Sulfuric Acid-Method-Based Developed Biosensor for DNA or RNA Target Detection

The developed biosensor is based on a series of steps involving the activation and functionalization of polystyrene 96-well microplates, as described in Scheme 1B and as will be detailed in the next paragraphs.

#### 2.4.1. Activation and Functional Modification of 96-Well Microplate Surfaces

In order to facilitate the binding of untreated single-stranded DNA, the surface of a polystyrene microplate was modified by utilizing the amine groups present in its nitrogenous bases. This process involved two main phases to modify each well of the microplate. Initially, the activation of the plate surface involved treating the wells with potassium hydroxide (KOH) for 10 min, employing a total volume of 100 µL. This activation step facilitated the generation of hydroxyl groups, which were crucial for subsequent silanization. Following this, the microplates underwent thorough rinsing with distilled water, ensuring a minimum of three wash cycles. Subsequently, 100 µL of APTES was introduced into each well to initiate the process of silanization. During this silanization process, silanol linkages (-O-Si-) were established between the hydroxyl functional groups (-OH) on the plate’s surface and the alkoxy groups present in APTES, thus introducing amino functionalities. After 30 min of silanization, the amino-functionalized polystyrene 96-well microplates were subjected to three washes with 10 mM of PBS. As a result, the substrate of the polystyrene 96-well microplate was effectively altered to incorporate amine functionality (Scheme 1(Ba)).

#### 2.4.2. Immobilizing Probe on the Amine-Functionalized 96-Well Microplate

To immobilize the unmodified ssDNA probe complementary to the RNA or DNA target, glutaraldehyde was utilized as a cross-linking agent. Glutaraldehyde was immobilized on the NH_2_-functionalized polystyrene 96-well microplate surface, forming a C=N linkage. The reaction took place over 45 min, utilizing a suitable concentration of 5%. Subsequently, the microplate underwent rinsing with distilled water. Then, 1 µM of the capture probe (ssDNA) in 10 mM of PBS at pH 7.4 was introduced into each well, allowing an hour of incubation at 37 °C. After three washes, non-specific sites were obstructed for 15 min using 1 mM of ethanolamine. The outcome was a modified microplate carrying the capture probe (DNA_capture_-modified microplate), acting as a pre-activated bio-platform ready-to-use for on-site identification of DNA or RNA targets (Scheme 1(Bb)).

#### 2.4.3. Target RNA/DNA Hybridization

For target DNA or RNA detection, strict hybridization conditions were maintained. A volume of 100 µL containing target DNA or RNA at various concentrations (ranging from 0.2 µM to 1 µM) in PBS was added to each DNA_capture_-modified plate. The hybridization process was allowed to proceed for one hour at a temperature of 37 °C. Subsequently, the wells were thoroughly washed three times using a washing buffer, ensuring the removal of any non-specifically bound molecules (Scheme 1(Bc)).

#### 2.4.4. Target DNA/RNA Detection via Phenol–Sulfuric Acid Method

To initiate the reaction, 100 µL of concentrated sulfuric acid and 10 µL of phenol (120 mg/mL) were added to each well (DNA_capture_-modified microplate), before and after hybridization step. A yellow-orange color was immediately developed in the solution. Subsequently, the color produced was measured at a wavelength of 490 nm using an ELx800 absorbance microplate reader (Scheme 1(Bd)).

### 2.5. Construction of a Biosensor for Intercalating Agent Detection Based on the Phenol–Sulfuric Acid Method

The biosensor was constructed following the steps outlined above, including activation, functionalization, and DNA capture immobilization. During the hybridization step, a solution containing the DNA target (1 µM) and Curcumin at different concentrations (ranging from 10 µM to 100 µM) was added simultaneously. The solution was incubated for 1 h at 37 °C. Subsequently, a washing step was performed to remove any of the unhybridized DNA target. Notably, the presence of Curcumin demonstrated an inhibitory effect on the hybridization process. Then, the developed method based on the phenol and sulfuric acid described above was carried out to evaluate the concentration of Curcumin.

## 3. Results and Discussions

### 3.1. Integrated Approach for Sugar Source Analysis and DNA Detection Using Microplate-Based Phenol–Sulfuric Acid Method

The reaction between sugars and phenol–sulfuric acid is based on the condensation of hydroxyl groups in sugars with phenol groups in the presence of sulfuric acid. This condensation reaction forms furfurals, which exhibit strong absorption at 490 nm (Figure S1) [1,28]. The sulfuric acid serves as a dehydrating agent, facilitating the conversion of sugars into furfurals. The yellow-orange color intensity is directly proportional to the sugar concentration in the sample. This method provides a reliable and sensitive approach for sugar source analysis, enabling accurate determination of sugar content in various samples.

#### 3.1.1. Sugar Reaction via the Developed Phenol–Sulfuric Acid Method

**Figure 1:**
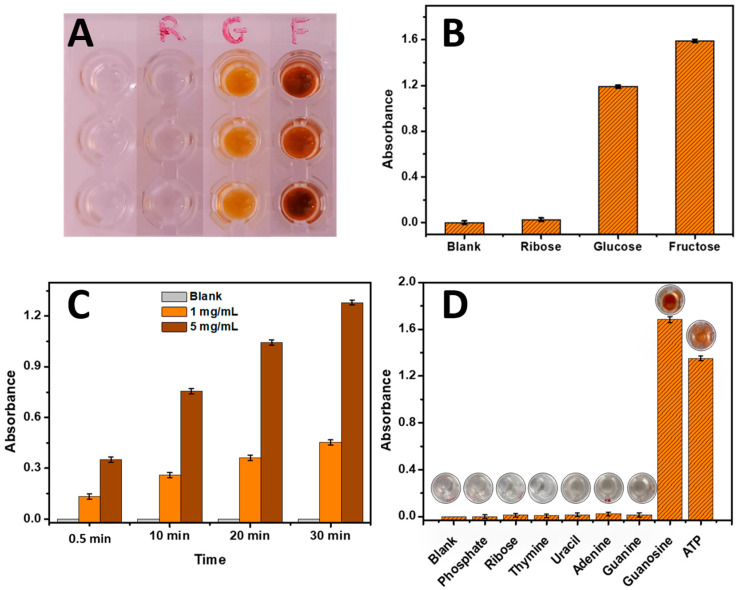
(**A**) Image of the yellow-orange produced color from the phenol–sulfuric acid reaction with (10 mg/mL) ribose, glucose, and fructose; (**B**) product absorbance of phenol–sulfuric acid reaction with (10 mg/mL) ribose, glucose, and fructose measured at 490 nm. (**C**) Absorbance of DNA fish sperm (1 mg/mL and 5 mg/mL) reacted with phenol–sulfuric acid at different incubation times (0.5 min, 10 min, 20 min, and 30 min) measured at 490 nm. (**D**) Absorbance histograms of DNA components (phosphate, ribose, thymine, uracil, adenine, guanine, and guanosine) and ATP using the phenol–sulfuric acid method at 490 nm. Error bars were obtained from three parallel experiments.

Three sugars, namely Ribose, Glucose, and Fructose, were investigated to evaluate the phenol–sulfuric acid reaction. The concentrations used were 10 mg/mL for each sugar. The results are presented in Figure 1. As depicted in Figure 1A and confirmed in Figure 1B, the blank control without any sugar source remained colorless, indicating the absence of a reaction. However, the wells containing Ribose exhibited a mild color change, consistent with the literature findings that Ribose yields a weaker signal compared to Glucose and Fructose [3]. In contrast, the Glucose and Fructose wells displayed intense coloration, confirming their higher reactivity with the phenol–sulfuric acid reagent. This observation aligns with previous studies highlighting the different reactivity levels among these sugars [3].

#### 3.1.2. Application of the Phenol–Sulfuric Acid Sugar Reaction Method for DNA of Fish Sperm Analysis

Since the reaction of sugars with phenol–sulfuric acid was effective, it was employed for the analysis of DNA from fish sperm samples, considering that DNA contains ribose as a sugar source. DNA samples from fish sperm were analyzed at different concentrations (0, 1, and 5 mg/mL). The results are presented in Figure 1C. It can be observed that as the concentration of DNA fish sperm increased from 0 mg/mL to 5 mg/mL, the absorbance of the resulting color also increased. This increase in absorbance cannot be attributed only to the higher concentration of ribose present in the DNA samples, since the absorbances obtained with free ribose (Figure 1B) are lower than the absorbances obtained with ribose as a key sugar component of DNA (Figure 1C). Additionally, it is noteworthy that the presence of sulfuric acid in the reaction mixture leads to a slight hydrolysis of the DNA strands, contributing to the generation of nucleosides, which may lead to high color intensity. Furthermore, as described in the literature, the absorbance showed a time-dependent increase for all concentrations (Table S1).

#### 3.1.3. Application of the Phenol–Sulfuric Acid Sugar Reaction Method for Each Component of DNA

In this study, various DNA compounds including phosphate, thymine, uracil, guanine, adenine, ribose, guanosine, and ATP were individually analyzed using the phenol–sulfuric acid reaction method. As shown in Figure 1D, the results revealed that except for guanosine and ATP, none of the other compounds exhibited significant absorbance. This surprising outcome suggests that the phenol–sulfuric acid reaction specifically interacts with the sugar component present in guanosine and ATP. The reactivity towards guanosine and ATP indicates the capability of the phenol–sulfuric acid reaction to react with ribose-based compounds. These findings highlight the importance of understanding the chemical reaction of the phenol–sulfuric acid method and its potential applicability as a method for the quantification of DNA. This opens up potential avenues for developing sensitive and specific assays for molecular diagnostics and genetic analysis. For that, this research focuses on refining and optimizing the phenol–sulfuric acid method to expand its scope and enhance its utility in DNA biosensing applications.

### 3.2. DNA-Based Biosensor for Specific Targets (DNA and RNA) Detection Using the Phenol–Sulfuric Acid Method

**Figure 2:**
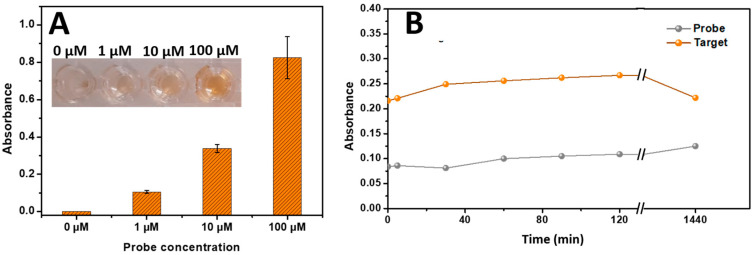
(**A**) Absorbance histograms of immobilized DNA_Probe_ at various concentrations (0 µM, 1 µM, 10 µM, and 100 µM) using the phenol–sulfuric acid reaction on a 96-well microplate at 490 nm. (**B**) Kinetic curves of the detection step on the phenol–sulfuric acid-reaction-based developed biosensor in the absence and presence of the target sequence. The absorbance was measured at 490 nm. Error bars were obtained from three parallel experiments.

The immobilization of the unmodified probe was successfully demonstrated by immobilizing various concentration of probe (1 µM, 10 µM, and 100 µM) on the well surface and subjected the phenol–sulfuric acid reaction. As described above in Figure 2A, the absorbance in the wells increased with the increase in the probe concentration immobilized on the wells.

Using the phenol–sulfuric acid method combined with the DNA_capture_-modified microplate, specific targets (DNA and RNA) were analyzed. Once the DNA_capture_-modified plate has been established as described above (Scheme 1B), in the presence of the target DNA or RNA, the capture probe hybridized with the complementary sequence, forming a double-stranded structure. The concentration of the target DNA or RNA captured by the capture probe was quantified using the phenol–sulfuric acid method. The ribose/deoxyribose component present in the backbone of DNA or RNA reacted with the phenol and the sulfuric acid, resulting in the development of a colored complex measured at 490 nm. The higher the concentration of the target DNA or RNA, the higher the level of the colored complex generated (Figure 2A). This principle enables the quantification of the target DNA or RNA using the developed biosensor, offering a potential tool for sensitive and specific detection in various applications.

The time-dependent behavior of the proposed biosensor was investigated to assess the reaction kinetics for target DNA or RNA detection. As illustrated in Figure 2B, the absorbance exhibited an instantaneous high increase in absorbance followed with a very slight increase as a function of time, indicating that the color development process did not significantly impede the measurement of absorbance, which can be performed the first minute or several hours later. This finding suggests that the biosensor can be effectively monitored within the first minute. By measuring the absorbance during this early stage, accurate and time-efficient detection can be achieved, streamlining the overall assay procedure.

### 3.3. Optimizing Phenol and Sulfuric Acid Concentration Parameters for the Developed Biosensor Based on the Phenol–Sulfuric Acid reaction

**Figure 3:**
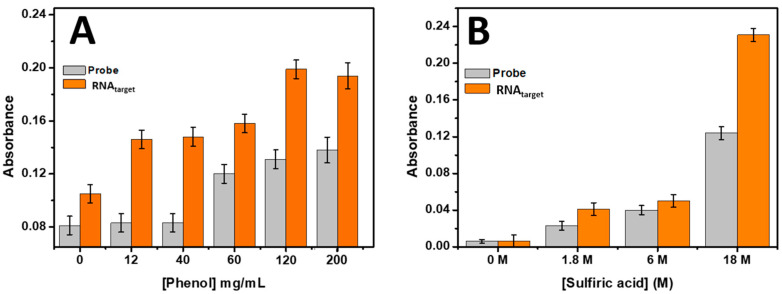
Optimization of (**A**) phenol concentration (from 0 to 200 mg/mL) and (**B**) sulfuric acid concentration (from 0 to 18 M) induced in the reaction of phenol–sulfuric acid. The absorbance was measured at 490 nm. Error bars were obtained from three parallel experiments.

The optimization of reagent concentrations, specifically sulfuric acid and phenol, was conducted to determine the optimal conditions for detecting 0.5 µM of target RNA using the proposed biosensor. Various concentrations of phenol ranging from 0 to 200 mg/mL were tested in combination with concentrated sulfuric acid. The results, presented in Figure 3A, revealed that the absorbance increased with the increase in phenol concentration, reaching a maximum at 120 mg/mL. Based on this finding, which agrees with the literature [1,2,3] and takes into account the concept of green analytical chemistry, a phenol concentration of 120 mg/mL was chosen for subsequent experiments.

Furthermore, the concentration of sulfuric acid was also optimized by testing concentrated sulfuric acid, as well as dilutions of 3 times and 10 times, while keeping the phenol concentration fixed at 120 mg/mL for 0.5 µM of target RNA. The results (Figure 3B) demonstrated that most concentrated sulfuric acid yielded the highest absorbance response in the developed biosensor. Therefore, concentrated sulfuric acid was selected as the optimal concentration for the rest of the study.

The optimization of both sulfuric acid and phenol concentrations ensures the maximum absorbance response in the biosensor, enhancing its sensitivity and accuracy for target RNA detection. These findings provide valuable insights into the selection of appropriate reagent concentrations to optimize the performance of the proposed biosensor.

### 3.4. Application of the Developed DNA-Based Biosensor for DNA or RNA Target Detection

#### 3.4.1. Analytical Performance

The analytical performance of the developed biosensor was evaluated under the optimized conditions. Different concentrations of target DNA or RNA were incubated in separate microplates and subjected to the proposed detection method, resulting in a visible color change. The color intensity gradually increased as the concentration of target DNA or RNA increased from 0.2 µM to 1 µM.

**Figure 4:**
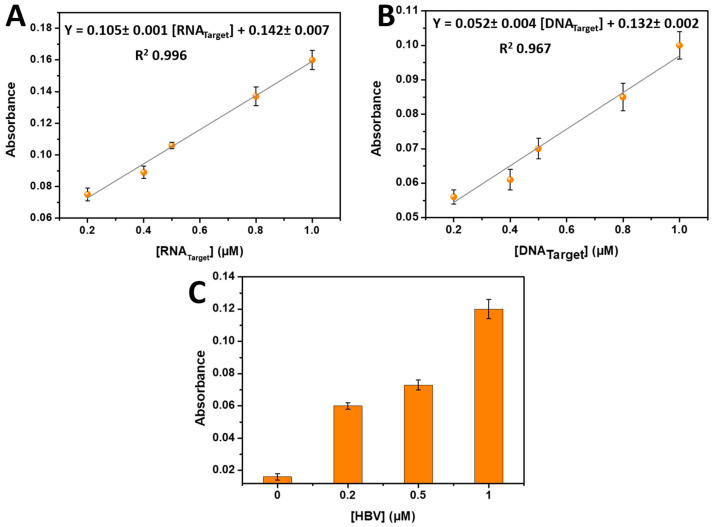
(**A**) Calibration plots of absorbance values measured at 490 nm against different microRNA-21 target concentrations ranging from 0.2 µM to 1 µM. (**B**) Calibration plots of absorbance values measured at 490 nm as a function of different concentrations of DNA target from 0.2 µM to 1 µM. (**C**) Application of the developed biosensor for 0, 0.2, 0.5, and 1 µM of HBV. Three parallel experiments yielded error bars.

Figure 4A,B demonstrate that a linear relationship exists between the DNA target concentrations ranging from 0.2 µM to 1 µM and the RNA target concentrations ranging from 0.2 µM to 1 µM. This linear relationship confirms the quantitative nature of the biosensor’s response to varying target concentrations. Moreover, the limit of detection (LOD) for RNA detection was estimated to be 0.17 µM, while the LOD for DNA detection was determined to be 0.2 µM at a signal/noise ratio of 3.

The linear regression equations were derived as follows: Y = 0.142 ± 0.007 + 0.105 ± 0.001 [RNA_Target_] (µM) with a correlation coefficient (R^2^) of 0.996 for RNA detection, and Y = 0.132 ± 0.002 + 0.052 ± 0.004 DNA target (µM) with an R^2^ value of 0.967 for DNA detection. These regression equations highlight the linear relationship between the biosensor’s response and the concentrations of the target DNA or RNA.

Overall, the developed biosensor exhibits excellent analytical performance with a low limit of detection and a strong correlation between target concentration and color intensity. The linear range obtained meets the concentration values of the extracted DNA or RNA from the well-established kits [29]. These results demonstrate the potential of the biosensor for sensitive and accurate detection of target DNA or RNA in various applications. For that and as an application, the developed biosensor was tested with various concentrations of hepatitis B virus (HBV) as the DNA target. The DNA capture probe was immobilized on the microplate, and different concentrations of the HBV target (0, 0.2, 0.5, and 1 µM) were added. After the hybridization step, phenol and sulfuric acid were introduced to the wells, and the absorbance was measured at 490 nm. The results are presented in Figure 4C, clearly indicating that the absorbance increased with higher HBV DNA target concentrations, confirming the successful application of the developed biosensor for HBV detection.

#### 3.4.2. Selectivity

**Figure 5:**
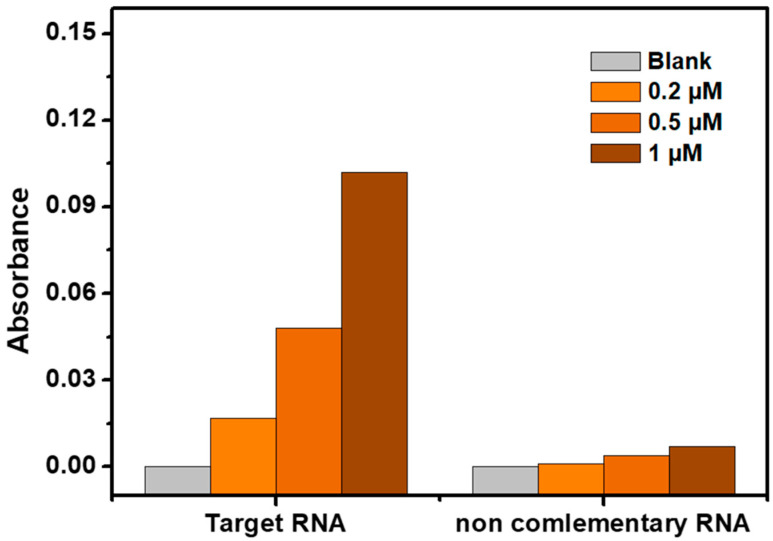
Selectivity study of the developed phenol–sulfuric acid-based biosensor toward 0, 0.2, 0.5, and 1 µM of noncomplementary RNA target.

To assess the selectivity of the developed biosensor for RNA target detection, a selectivity test was conducted using a perfectly non-complementary target RNA at different concentrations (0, 0.2 µM, 0.5 µM, and 1 µM). The results, depicted in Figure 5, clearly demonstrate that the absorbance of the colorimetric assay towards the perfectly matched target RNA was significantly stronger compared to the non-complementary target RNA at all concentrations. This observation highlights the high selectivity of the proposed method for RNA target detection. The sensitivity and selectivity combination of the biosensor make the proposed biosensor a promising tool for the quantitative detection of targets. Overall, the results of the selectivity study support the conclusion that the developed biosensor offers a sensitive, selective, and simple approach with a high quantitative capacity for the detection of RNA/DNA target sequences.

### 3.5. Application of the DNA-Based Biosensor Developed for the Detection of Intercalating Agents

**Figure 6:**
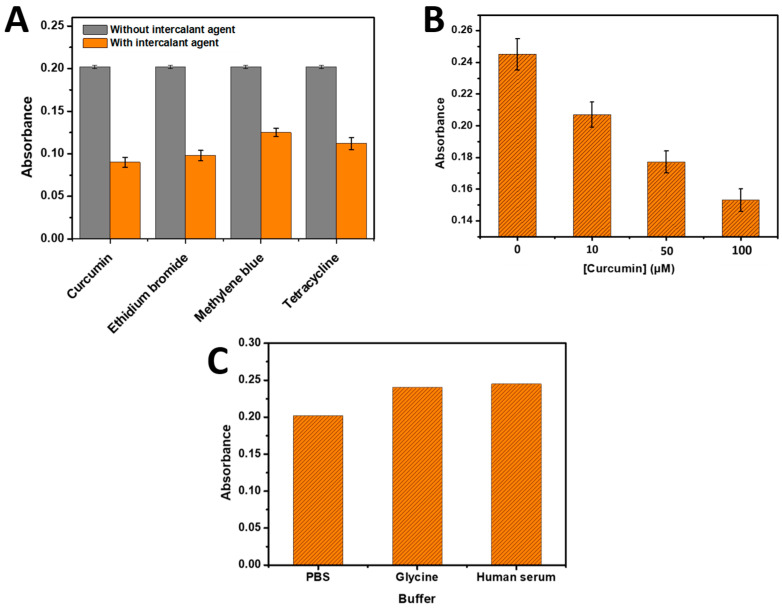
(**A**) Absorbance histograms of phenol–sulfuric acid-based developed bioassays in the absence and presence of 100 µM of various intercalating agents (Methylene blue, Curcumin, ethidium bromide, and tetracycline) at 490 nm. (**B**) Absorbance histograms of various Curcumin concentration (0, 10, 50, and 100 µM) tested in the developed bioassays. Three parallel experiments yielded error bars. (**C**) Absorbance histograms of phenol–sulfuric acid developed biosensor with 0.5 µM of target DNA in different buffers.

**Scheme 2:**
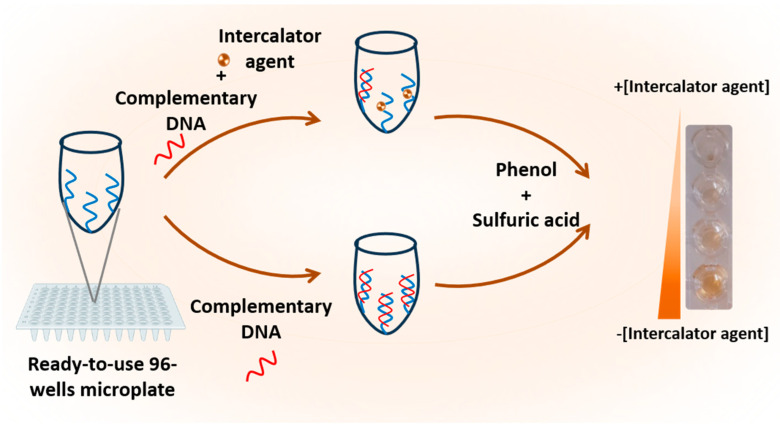
Illustration of the colorimetric biosensor based on the phenol–sulfuric acid reaction for intercalator agent detection.

The versatility of the developed biosensor was further demonstrated by its application in detecting intercalating agents that hinder the hybridization of DNA complementary to the immobilized capture DNA on the 96-well microplate (Scheme 2). To evaluate the inhibition of the hybridization step of the developed bioassay for an intercalating agent detection, several intercalating agents were employed. Figure 6A illustrates the results obtained with (100 µM) methylene blue, Curcumin, ethidium bromide, and tetracycline, respectively, which were tested for their ability to inhibit the hybridization reaction (using 0.5 µM of complementary RNA) and subsequently measured using the developed bioassay. All intercalating agents exhibited a decrease in absorbance upon their addition, indicating an inhibitory effect on the hybridization step. This decrease in signal can be attributed to the interference caused by the intercalating agents during the hybridization process. The Curcumin and ethidium bromide showed the most inhibitor agents in the present bioassay.

#### Application for Curcumin Intercalating Agent

The developed biosensor was also employed for the quantification of Curcumin as the intercalant agent that inhibited the hybridization of DNA complementary to the immobilized capture DNA on the 96-microplate wells. Curcumin, a commonly used intercalant agent, was chosen for this application. Different concentrations of Curcumin solution (10 µM, 50 µM, and 100 µM) were added simultaneously with the target DNA (0.5 µM), which resulted in an initial absorbance of 0.240. The presence of Curcumin hindered the hybridization process, leading to a decrease in the absorbance obtained from the developed method based on the phenol–sulfuric acid detection.

As illustrated in Figure 6B, the absorbance gradually decreased with an increase in Curcumin concentration. This indicates that higher concentrations of Curcumin resulted in a greater inhibition of the hybridization process between the DNA target and the immobilized capture DNA. The inhibitory effect of Curcumin on the hybridization process can be attributed to its intercalation between DNA base pairs, which disrupt the stability of the double-stranded DNA structure.

Furthermore, the hybridization step with 0.5 µM of target DNA was performed in glycine and commercial human serum. As shown in Figure 6C, the resulting absorbance for both glycine and human serum was comparable to that obtained in the PBS buffer, suggesting the absence of intercalating agents in glycine and human serum that could hinder the hybridization step. This finding is supported by the technical specifications of the commercial human serum from Sigma-Aldrich, which indicates the composition of the prepared human serum (210 mg/dL of cholesterol, 175 mg/dL of triglyceride, 140 mg/dL of glucose, 9.0 g/dL of total protein, and <30 mg/dL of hemoglobin).

The successful detection and quantification of intercalant agents using the developed biosensor highlight its versatility and potential applications in the field of DNA genosensor analysis. By incorporating intercalant agent detection into the biosensor platform, it becomes a valuable tool for studying the interactions between intercalant agents and DNA molecules, as well as for screening potential intercalators or investigating the effects of intercalation on DNA hybridization efficiency.

Overall, the results obtained in this study demonstrate the capability of the developed biosensor to evaluate the genotoxicity of the intercalant agents, offering a new avenue for applications in drug development, environmental monitoring, and genetic research.

## 4. Conclusions

In conclusion, the development of the biosensor utilizing the phenol–sulfuric acid reagent for sugar detection was successfully employed for the detection of DNA and RNA, as specific target and intercalator molecules, thanks to the significant color intensity of the nucleoside produced under partial hydrolysis with sulfuric acid at room temperature. With its advantages of simplicity, cost-effectiveness, and potential for further optimization, the proposed biosensor paves the way to various applications in molecular diagnostics, genetic analysis, and drug discovery. The successful application of this biosensor to detect DNA, RNA, and intercalator molecules serves as a compelling proof of concept for its capabilities and effectiveness as a bioanalytical tool.

## Figures and Tables

**Table 1:** Nucleic acids utilized in the present work.

Nucleic Acid	Sequence (5′-3′)
Target microRNA-21	5′-UAGCUUAUCAGACUGAUGUUGA-3′
DNA_probe_ (complementary sequence of microRNA-21)	5′-AAATCAACATCAGTCTGATAAGCTA-3′
Target DNA (*E. coli*)	5′-TATTAACTTTACTCCCTTCCTCCCCGCTGA-3′
DNA_probe_ (complementary sequence of DNA_*E. coli*_)	5′-TCAGCGGGGAGGAAGGGAGTAAAGTTAATA-3′
MicroRNA-155 (non-complementary oligonucleotide)	5′-UUAAUGCUAAUCGUGAUAGGGGUU-3′
Target DNA (Hepatitis B virus HBV)	5′-GTGTCTGCGGCGTTTTATCATCTTC-3′
DNA_probe_ (complementary sequence of DNA_HBV_)	5′-TGATAAAACGCCGCAGACAC-3′

## Supplementary Materials

The following supporting information can be downloaded at https://www.mdpi.com/article/10.3390/bios13090853/s1, Figure S1: spectrum of formed complex using the phenol–sulfuric acid method with sugar, exhibiting maximum absorbance at 490 nm; Table S1: images of DNA fish sperm (1 mg/mL and 5 mg/mL) reacted with phenol–sulfuric acid after 0.5 min, 10 min, 20 min, and 30 min of reaction incubation time.
