# Supplementary material for: Development of an Innovative Colorimetric DNA Biosensor Based on Sugar Measurement
Source: Biosensors (Basel). 2023 Aug 28;13(9):853. doi: 10.3390/bios13090853 (PMC10526849; doi:10.3390/bios13090853)
Supplement: Supplementary file 1 [file biosensors-13-00853-s001.zip › biosensors-2566762-supplementary.pdf]

biosensors

Supplementary Materials

Development of an innovative colorimetric DNA Biosensor based on sugar measurement

Maliana El Aamri, Yasmine Khalki, Hasna Mohammadi and Aziz Amine *

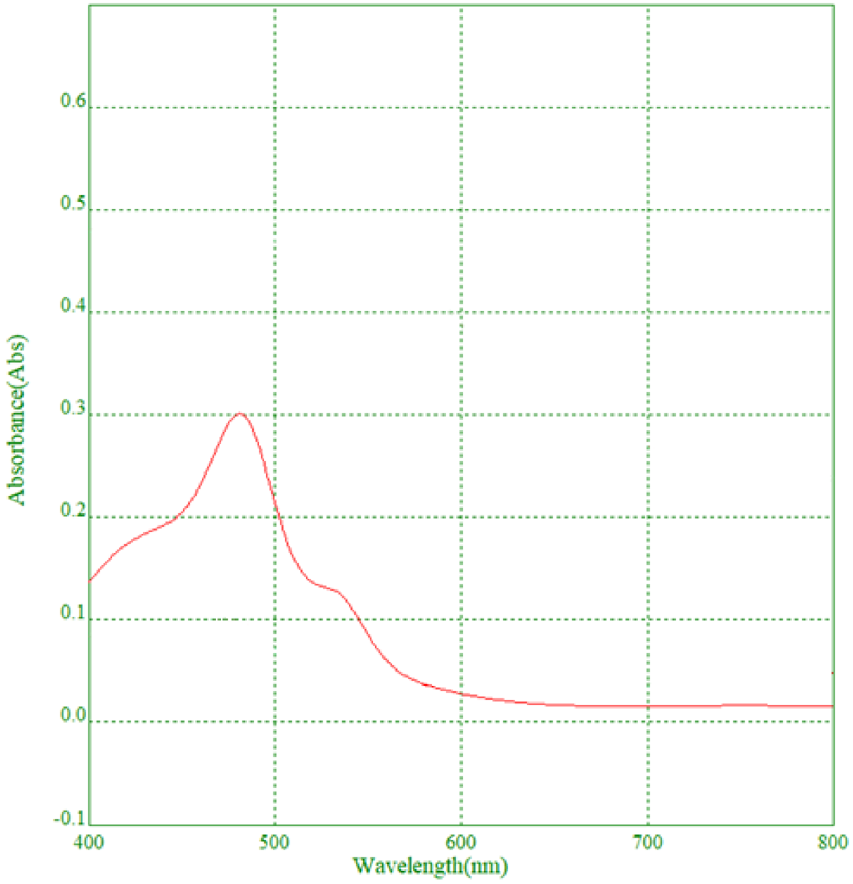

Figure S1: Spectrum of Formed Complex Using the Phenol-Sulfuric Acid Method with Sugar, Exhibiting Maximum Absorbance at 490 nm.

Table S1. Images of DNA fish sperm (1 mg/mL and 5 mg/mL) reacted with phenol - sulfuric acid after 0.5 min, 10 min, 20 min and 30 min of reaction incubation time.

	**0 mg/mL**	**1 mg/mL**	**5 mg/mL**
0.5 min	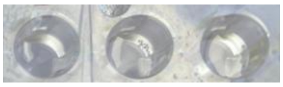	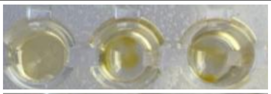	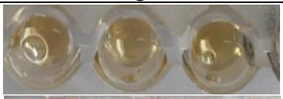
10 min	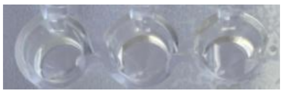	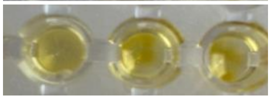	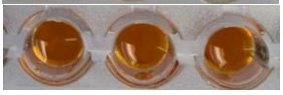
20 min	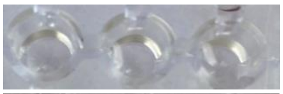	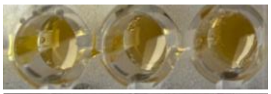	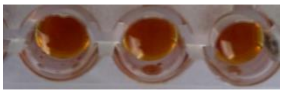
30 min	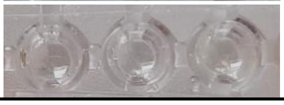	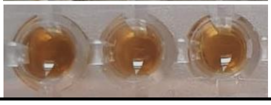	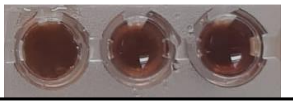
